# X-ray diffraction with micrometre spatial resolution for highly absorbing samples

**DOI:** 10.1107/S1600577522008025

**Published:** 2022-10-05

**Authors:** Prerana Chakrabarti, Anna Wildeis, Markus Hartmann, Robert Brandt, Ralph Döhrmann, Giovanni Fevola, Christina Ossig, Michael Elias Stuckelberger, Jan Garrevoet, Ken Vidar Falch, Vanessa Galbierz, Gerald Falkenberg, Peter Modregger

**Affiliations:** a Physics Department, University of Siegen, 57072 Siegen, Germany; b Center for X-ray and Nano Science CXNS, Deutsches Elektronen-Synchrotron DESY, 22607 Hamburg, Germany; c Mechanical Engineering Department, University of Siegen, 57076 Siegen, Germany; d Physics Department, University of Hamburg, 22761 Hamburg, Germany; e Deutsches Elektronen-Synchrotron DESY, 22607 Hamburg, Germany; University of Tokyo, Japan

**Keywords:** X-ray diffraction, high spatial resolution, high photon energy, X-ray fluorescence, goniometers

## Abstract

X-ray diffraction with high spatial resolution is commonly used to characterize (poly)crystalline samples with, for example, respect to local strain, residual stress, grain boundaries and texture. However, the investigation of highly absorbing samples or the simultaneous assessment of high-*Z* materials by X-ray fluorescence have been limited due to the utilization of low photon energies. Here, a goniometer-based setup implemented at the P06 beamline of PETRA III that allows for micrometre spatial resolution with a photon energy of 35 keV and above is reported. A highly focused beam was achieved by using compound refractive lenses, and high-precision sample manipulation was enabled by a goniometer that allows up to 5D scans (three rotations and two translations). As experimental examples, the determination of local strain variations in martensitic steel samples with micrometre spatial resolution, as well as the simultaneous elemental distribution for high-*Z* materials in a thin-film solar cell, are demonstrated. The proposed approach allows users from the materials-science community to determine micro-structural properties even in highly absorbing samples.

## Abstract 2

A demonstration of high-resolution micro X-ray diffraction at high photon energies for highly absorbing samples.

## 1. Introduction

Local deformations of (poly)crystalline structures, such as strain, tilt or grain boundaries, can have a profound impact on the performance of functional and electronic materials. Two examples are the conversion efficiency of solar cells or the fatigue strength in spring steels. High-resolution micro X-ray diffraction (µ-HRXRD) utilizes focused X-ray beams to study local defects on the nanoscale.

Examples of µ-HRXRD include the study of local microstrain and tilt distribution in CuInSe_2_ functional thin films with average grain sizes below 1 µm, a photon energy of 8.9 keV, a spot size of 100 nm × 100 nm and a strain sensitivity of the order of 10^−4^ at the ID01 beamline of the European Synchrotron Radiation Facility (Schäfer *et al.*, 2016 ▸). A similar characterization of CdTe crystallites with sub-100-nm grain sizes occurs in fully operational photovoltaic cells with similar experimental parameters at the hard X-ray nanoprobe (HXN) beamline 3-ID of the National Synchrotron Light Source II (Calvo-Almazan *et al.*, 2019 ▸). Another example is the determination of local microstructure of martensite-retained austenite steel utilizing a photon energy of 12 keV and a spot size of 4 µm × 1 µm at the 2-ID-D undulator beamline of the Advanced Photon Source (Cai *et al.*, 2001 ▸), where the grain size was found to be much smaller than the focal spot size. A final example is the renovated high-pressure XRD setup at the BL10XU beamline of SPring-8 (Hirao *et al.*, 2020 ▸) utilizing a photon energy of 30 keV and a spot size of 1 µm × 1 µm, where the angular sensitivity – conservatively estimated as the ratio between pixel size and sample-to-detector distance – was ∼2.2 × 10^−4^ rad and the maximal sample thickness without compromising angular resolution was ∼150 µm.

Investigations with high spatial resolution of highly absorbing samples rely on high photon energies and, simultaneously, small spot sizes. For highly absorbing samples, the X-ray fluorescence (XRF) signal from lighter elements will be absorbed leading to different applicable sample volumes. Here, we report on a novel goniometer-based setup implemented at the P06 beamline of PETRA III that enables µ-HRXRD with a few-micrometre spatial resolution at a photon energy of 35 keV. In addition, the high photon energy allows for the simultaneous acquisition of the XRF signal of elements up to iodine.

## 2. Experimental setup

All measurements presented here were obtained at the microhutch of beamline P06 of PETRA III at DESY, Hamburg (Falkenberg *et al.*, 2020 ▸). The incident photon energy of 35 keV was selected by a double-crystal monochromator. Compound refractive lenses (CRLs) were used to focus the beam to a spot size of 2.0 µm × 1.2 µm, which was measured by knife-edge scans of crossed gold wires. The focal distance of the CRLs was 660 mm measured from the pinhole exit (0.4 mm diameter) of the *N*_2_-rinsed CRL box, which leads to a longitudinal spot size of a few millimetres and a photon flux above 10^9^ at the focal position (Falkenberg *et al.*, 2020 ▸).

**Figure 1:**
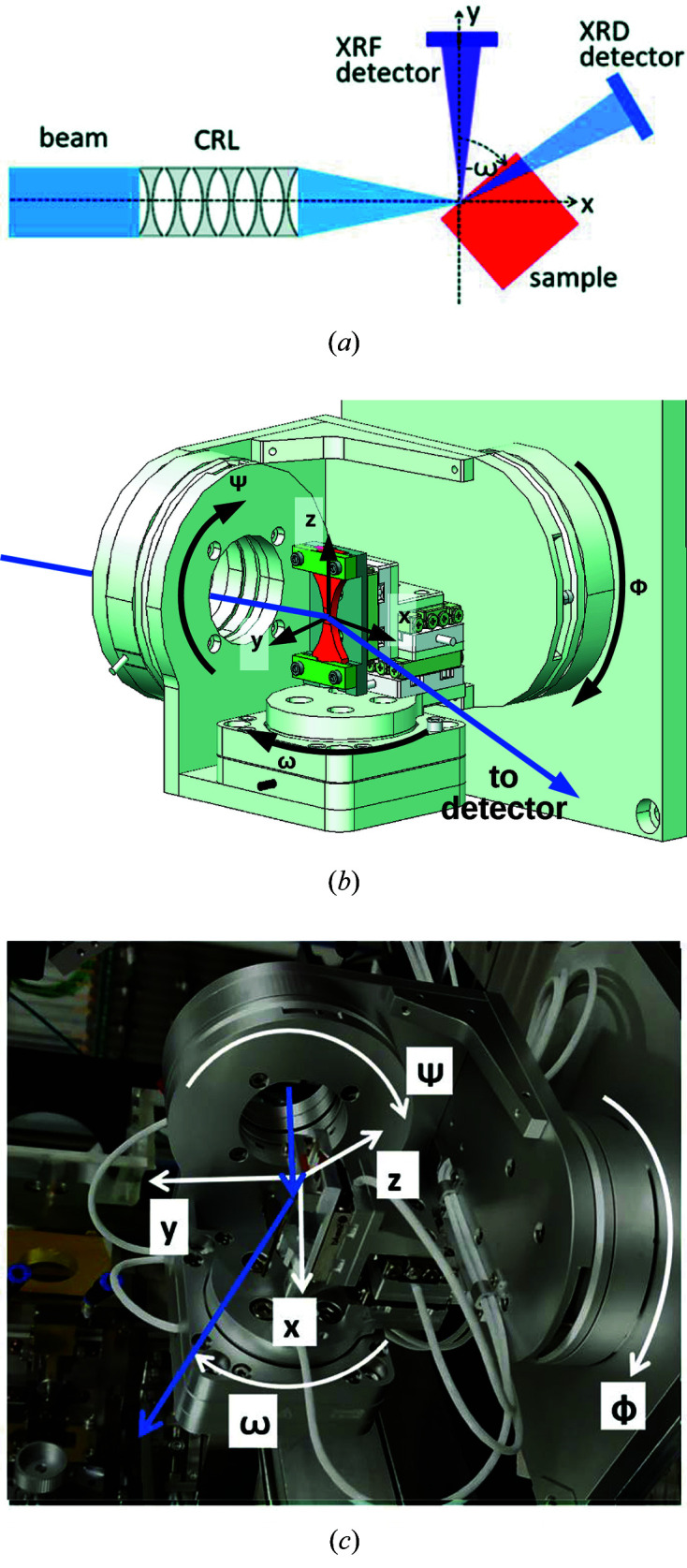
(*a*) The setup geometry (top view). (*b*) A technical drawing of the goniometer. The positioners are stacked from outer to inner in the order Φ, Ψ, ω, *y*, *x* and *z*. (*c*) A photograph of the goniometer.

A six-axes goniometer (SmarAct) fixed on a kinematic mount was used for sample manipulation [see Fig. 1 ▸(*b*)]. Fig. 1 ▸(*c*) shows the goniometer that was used for our experiment at P06, PETRA III. It featured three rotation circles with nominal angular resolutions below 0.1 µrad. Additional *x*, *y* and *z* translation stages on top of the last rotation enabled horizontal alignment along the beam (*x*) and scanning of the sample (*y* and *z*) with fixed rotation angles and a nominal position accuracy of 1 nm. Actual angular and positional accuracies have not yet been determined and upper bounds will be estimated below. The goniometer provides up to 5D scans for characterization of reciprocal space as a function of sample position. The XRD signal was obtained with a GaAs Lambda 2M detector (XSpectrum) with 55 µm pixel size, a 24-bit range and 1 ms readout frequency ∼1 m downstream of the sample and 25° from the incident beam [Fig. 1 ▸(*a*)]. In order to minimize absorption in the sample and, thus, to maximize detectable intensity, the incident beam impinged the sample through one side and exited through an orthogonal side. Thus, only a part of the diffraction pattern was measured, in contrast to the experiments listed in the *Introduction* [Sec sec1], which all utilized transmission geometry and measured the full Laue rings. The conservative estimate for the angular resolution of this setup is 5.5 × 10^−5^ rad, which implies a sensitivity to strain of 2.5 × 10^−4^ or better for the average 2θ of 25°. The maximum sample thickness without compromising angular resolution is ∼150 µm. The XRF signal was obtained using a Vortex silicon-drift detector (Hitachi High-Tech) located at 90° from the incident beam. The setup allowed photon energies of up to 42 keV.

The cross point of the rotation axes defines the desired scan position on the sample. Alignment of the cross point into the beam focus was achieved by a two-step procedure: first, an optical microscope was used to move the tip of a tomographic pin into the cross point and, second, the XRF signal of the pin was used to move the entire goniometer and, therefore, the cross point, into the beam focus.

## 3. Results

Two types of samples were scanned. Martensitic steel, as an example of a powder-like structure, as well as a thin-film solar cell, as an example of a single-crystal structure.

Martensite (*i.e.* α′-iron) is a meta-stable phase of carbon steel, which is distinguished by its micro- and nano-scopic lamellar structure (Krauss, 1999 ▸) resulting in a powder-like material system in the given context. Martensitic steels provide ultimate tensile strengths beyond 2 GPa and exhibit an excellent resilience in the high-cycle fatigue regime, which renders them as the material of choice for suspension springs in vehicles (Tump & Brandt, 2016 ▸). Martensite is produced by first heating specific iron–carbon alloys to temperatures of ∼1000°C, where austenite (*i.e.* γ-iron) with typical grain sizes of a few tens of nanometres is formed. Subsequently, the material is quenched, which prevents a diffusion of carbon atoms out of the crystal structure and allows the highly strained martensitic phase to form. During this process, martensite grows in a lamella-like structure in distinct directions within each prior austenite grain. The fatigue strength of martensitic steels can be further improved by introducing compressive residual stress via shot peening (Eleiche *et al.*, 2001 ▸), which constitutes the inducement of plastic deformation via striking the material surface with a blasting medium.

Here, we have used martensitic steel of type 54SiCr6, which was supplied in the form of wires with a diameter of 12 mm. Heat treatment consisted of austenization in vacuum at 1080°C for 100 min and gas quenching using compressed nitrogen, followed by tempering at 400°C for 1 h in an inert Ar atmosphere. Flat shallow-notched specimens were then manufactured by means of wire erosion. Subsequent shot peening was performed by using cast-iron steel-shot with an average size of 0.4 mm at 1.5 bar. Shot-peening intensities are commonly quantified by the Almen strip test, which involves the exposure of standardized steel strips identical to the considered shot-peening settings (Totten *et al.*, 2002 ▸). The arc height of the bending of the steel strips is then used to quantify the shot-peening intensity. Here, shot-peening intensities were 0.16 mm or 0.3 mm using one-sided peened steel strips.

The cross section of the martensite samples was quadratic with a side length of ∼0.7 mm, where the incident X-ray beam entered the sample through the front and exited through a side. The photon energy of 35 keV implied an absorption length of 250 µm. In the case where the diffraction condition was fulfilled throughout the beam path within the sample, its thickness implied a moderate broadening of the diffraction peaks of about seven pixels (compared with 20 pixel observed peak widths) and a corresponding decrease in angular sensitivity. The martensite samples were laterally scanned in the *y* and *z* directions with 51 by 51 steps of 2 µm each and an exposure time between 0.2 and 2 s. Upper bounds for the positional and angular accuracies of the goniometer were estimated by repeated movement to the same nominal values and cross checking the measured diffraction patterns. As no discernible deviations were observed, the positional accuracy was at least significantly smaller than the focus size (*i.e.* <1 µm) and the angular accuracy was significantly smaller than the numerical aperture (*i.e.* <6 × 10^−4^ rad).

**Figure 2:**
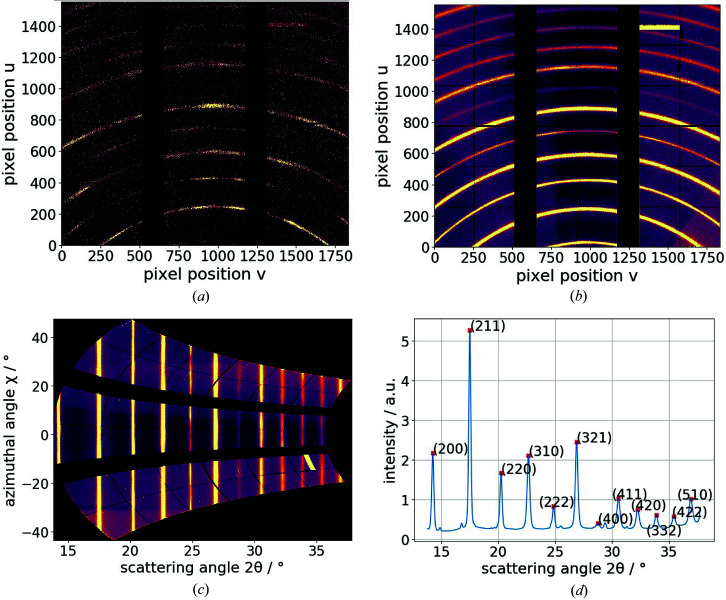
(*a*) A diffraction pattern of a single scan point of a martensitic steel sample. (*b*) A diffraction pattern of a martensitic steel sample summed over 2601 translation scan points. (*c*) Caking of the diffraction pattern in (*b*). (*d*) Final azimuthal integration of (*c*) yielding the 2θ positions of martensitic diffraction peaks. The yellow stripes in the upper right of (*b*) and the lower right of (*c*) are artefacts.

In order to translate pixel positions on the detector into 2θ values, we calibrated the setup geometry using the calibration routine provided by *PyFAI* (Ashiotis *et al.*, 2015 ▸; Kieffer & Karkoulis, 2013 ▸) and used lanthanium hexaboride (LaB_6_) as a diffraction standard. For automatic peak detection, all the diffraction patterns were summed up [Fig. 2 ▸(*b*)]. Single diffraction patterns [Fig. 2 ▸(*a*)] were transformed from Cartesian coordinates (*u*, *v*) to polar coordinates (2θ, χ) by so-called caking (or regrouping) (Kieffer & Karkoulis, 2013 ▸), and an example result is shown in Fig. 2 ▸(*c*). Subsequent vertical (*i.e.* azimuthal) integration provided the diffraction signal over 2θ. Fig. 2 ▸(*d*) shows that the first 12 martensitic diffraction peaks [*i.e.* from (200) to (510)] were detected. A slight texture can be seen in some of the Laue rings of the single scan points [Fig. 2 ▸(*a*)], but, in this proof-of-concept study these influences are neglected during azimuthal integration.

For the determination of angular position μ, angular width σ, height *H* and background *C* of all occurring diffraction peaks, the azimuthal integrated intensities were fitted to a Gaussian distribution (Zhang, 2011 ▸), given by 

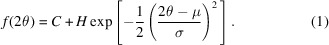

Local strain for the reflection *hkl*, ε_*hkl*_, is given by the differential Bragg equation (Hart, 1969 ▸),

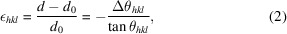

with *d* and *d*_0_ being the net plane spacing for strained and unstrained materials, respectively (Ramirez-Rico *et al.*, 2016 ▸). While martensite crystallizes in a body-centered tetragonal lattice and the *a*/*c* ratio depends, for example, on the carbon content (Sherby *et al.*, 2008 ▸), here we assume that *a*/*c* = 1, which yields a body-centred cubic structure. Furthermore, the lattice constant of unstrained martensitic steel was taken as *a* = 2.866 Å (Kim *et al.*, 2014 ▸). However, the sampling of the LaB_6_ calibration standard turned out to be insufficient (*i.e.* too few scan points), which resulted in an uncertainty in the determination of the sample–detector geometry. Thus, only relative strains will be reported here and an adequate number of scan points will be used in the future.

**Figure 3:**
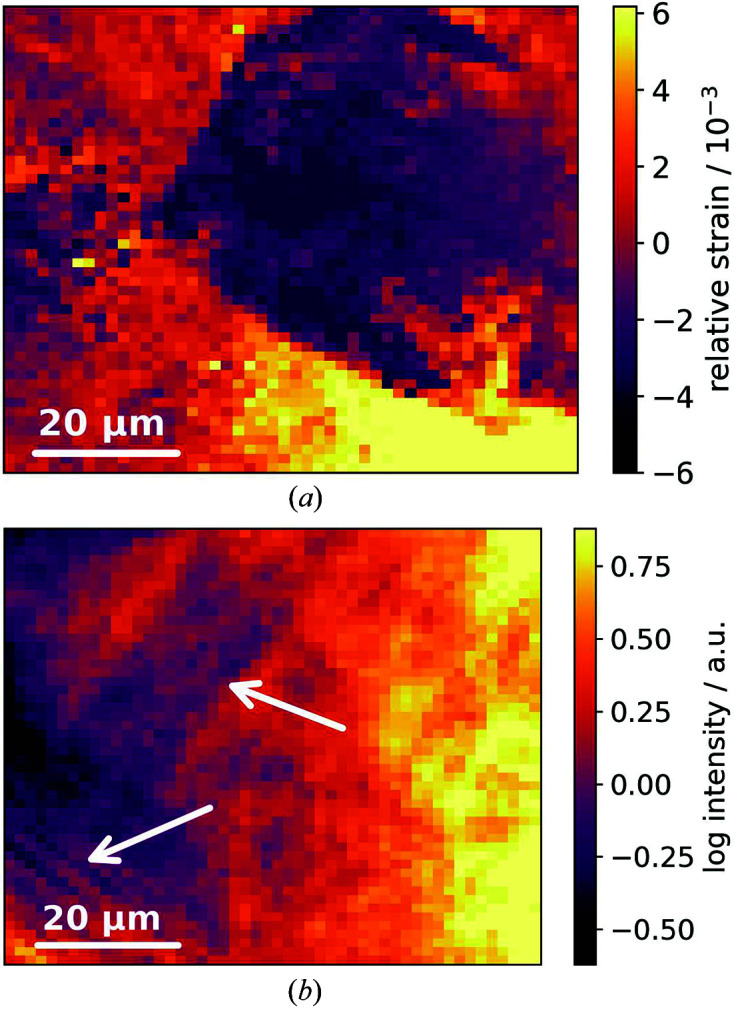
(*a*) Strain ε_200_ as a function of sample according to equation (2). (*b*) Intensity [*C* + *H* in equation (1)] of the (310) reflection showing lamella-like martensite structure. Although the images show the same area of the sample, the visible information relating to the two different reflections is complementary and originates from different structures.

Fig. 3 ▸(*a*) shows the relative strain in the [200] direction, ε_200_, as a function of position on the martensitic steel sample. The boundaries of one prior austenite grain are clearly visible. Fig. 3 ▸(*b*) shows the lamella-like structure of martensite in the intensity of the (310) reflection. The apparent dissimilarity between the images can be readily explained by the following. Due to the relatively large absorption length of 250 µm, martensitic structures of ∼10 to 20 randomly oriented prior austenite grains may contribute to the observable diffraction. Thus, the different reflections show different structures in Figs. 3 ▸(*a*) and 3 ▸(*b*).

**Figure 4:**
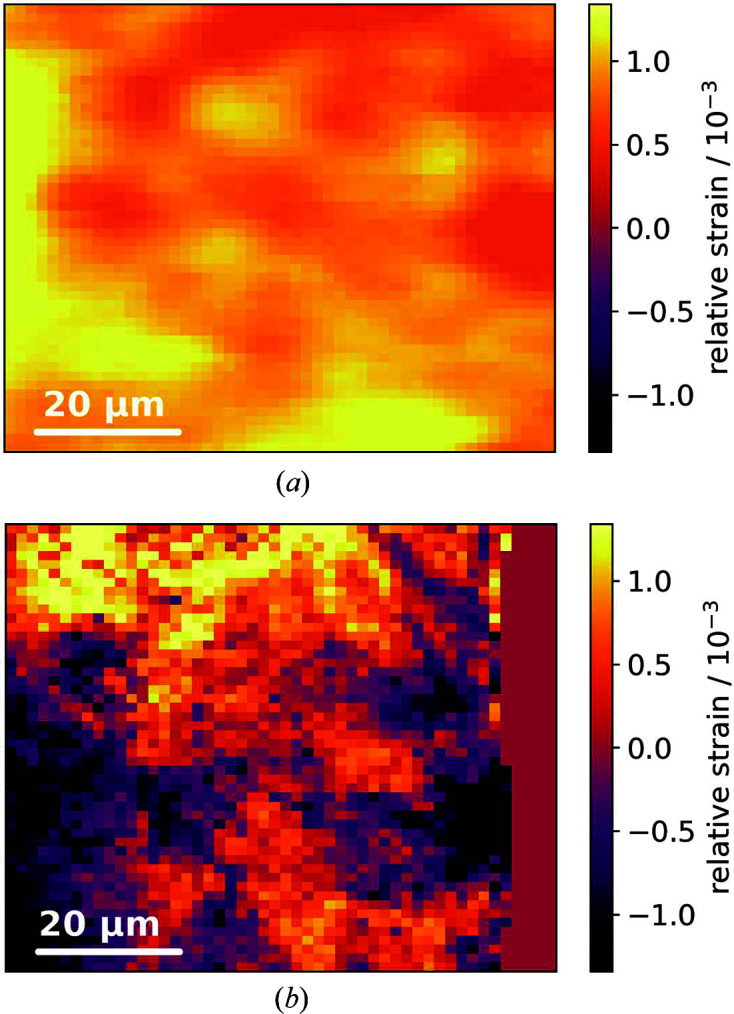
Relative strain maps in the [200] direction of two martensitic steel samples with a shot-peening Almen intensity of (*a*) 0.16 mm and (*b*) 0.3 mm. The images share the same colormap. The flat region on the right-hand side in (*b*) is out of the sample.

Fig. 4 ▸ compares the local relative strain in the [200] direction of two martensitic steel samples, which were treated with different shot-peening intensities as mentioned above. Although relative strains are displayed, the images are rooted in identical yet unknown absolute values, which allows for the utilization of the same colormap. As expected (Eleiche *et al.*, 2001 ▸), shot peening induces a compressive strain in the steel samples that scales with shot-peening intensity. Here, we measured an average compressive strain inducement of Δε_200_ = 8 × 10^−4^. At the same time we observed that increased shot-peening intensity also produces significant smaller microstructures. This visual impression is confirmed by the autocorrelation lengths of 37 µm for a shot-peening intensity of 0.16 mm [Fig. 4 ▸(*a*)] and 12 µm for 0.3 mm [Fig. 4 ▸(*b*)]. Out-of-beam-focus conditions for Fig. 4 ▸(*a*) were excluded by the fact that the goniometer was located on a kinematic mount and by the longitudinal spot sizes of a few millimetres. Furthermore, average peak counts in the analyzed reflections were 40000 for Fig. 4 ▸(*a*) and 17000 for Fig. 4 ▸(*b*).

Ultimately, these examples demonstrate the capability of µ-HRXRD at P06 for microstructural characterization for highly absorbing materials. For example, by taking advantage of the additional rotational degrees of freedom [Ψ and Φ in Fig. 1 ▸(*b*)], the full surface stress tensor of martensitic steel samples can be determined locally with micrometre spatial resolutions.

As an example of crystal structures providing well defined single Bragg peaks, we used a thin-film solar-cell sample. For these material systems, the combination of a microdiffraction goniometer with micro-XRF at high X-ray energies is compelling as it allows for a unique characterization of emerging photovoltaic materials. On the one hand, the accurate analysis of the elemental distribution in the compound absorber layer requires the excitation of *K*-shell electrons. On the other hand, elemental inhomogeneities in the polycrystalline semiconductors have been associated with strain-field variations and local solar-cell underperformance (Ulvestad *et al.*, 2019 ▸; Calvo-Almazan *et al.*, 2019 ▸; Correa-Baena *et al.*, 2019 ▸).

Here, we demonstrate an application example of a solar cell with an (Ag,Cu)(In,Ga)Se_2_ (ACIGS) absorber, where the local ratios of Ag/Cu (Aboulfadl *et al.*, 2021 ▸) and In/Ga (West *et al.*, 2017 ▸) that occupy the same lattice points are critical for the device performance. Moreover, the 50 nm-thick CdS layer forming the p–n junction together with the adjacent ACIGS and ZnO layers has been lacking spatial characterization in operational devices as the lateral distributions of Cd and In are only accessible with high-energy X-rays. The corresponding *K* absorption edges [*E*(Cd) = 27 keV and *E*(In) = 28.0 keV] are well accessible with the described setup.

The investigated solar cell was provided by Empa (Switzerland) and synthesized on soda–lime glass. The ACIGS absorber layer, which is the main interest, was grown on top of the Mo back electrode. Alkali metals including Na, K or Rb were added in a post-deposition treatment for the passivation of defects at grain boundaries. The front electrode consists of a CdS layer in contact with the absorber and an optically transparent ZnO absorber top electrode. For details of the synthesis conditions and sample properties, we refer to the description given by Yang *et al.* (2021 ▸).

For the simultaneous assessment of structure and composition employing X-ray diffraction and fluorescence, we mapped a 150 µm × 150 µm large area of an ACIGS solar cell in plan view with a step size of 1 µm × 1 µm. In the first approximation, the crystallites in the ACIGS material are randomly distributed and have a maximum size of a few micrometres. Accordingly, the Bragg condition is only sporadically fulfilled, which enables the unambiguous distinction of individual crystallites in real space that would form powder rings in classical diffraction measurements with larger beam size.

**Figure 5:**
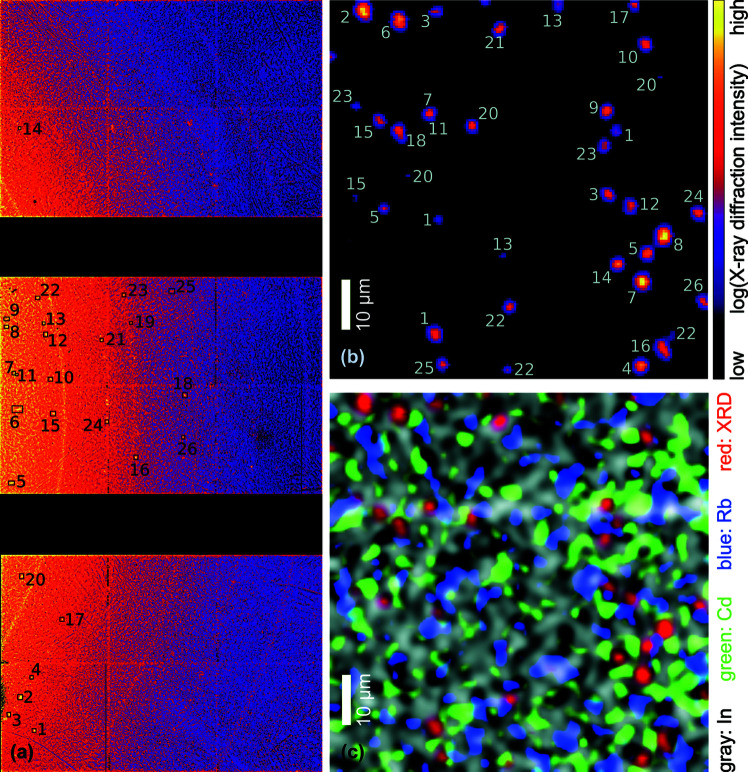
(*a*) An XRD detector image summed over the mapped area of a solar cell with a polycrystalline ACIGS absorber layer. The rectangles with adjacent numbers denote diffraction peaks from individual ACIGS crystallites. (*b*) A real-space map of the XRD intensity with the crystallites attributed to their position in the reciprocal-space image shown in (*a*). For example, locations in (*b*) indicated by ‘1’ provide the Bragg peak in (*a*) indicated by the same number. (*c*) A combined map of the XRD intensity (red) with the elemental distributions of In (gray), Cd (green) and Rb (blue).

The diffraction-detector images, integrated over the entire real-space map, are shown in Fig. 5 ▸(*a*). The peaks that could unambiguously be attributed to the ACIGS material are highlighted by black rectangles and enumerated for their localization in the real-space map shown in Fig. 5 ▸(*b*). The compositional variations within the ACIGS material and the tetragonal lattice lead to overlapping 2θ ranges from distinct lattice planes and pose a particular challenge for the conversion of the lattice spacing into strain; a detailed discussion of this aspect is beyond the scope of this article and will be addressed in a dedicated article about correlative nano­diffraction results.

**Figure 6:**
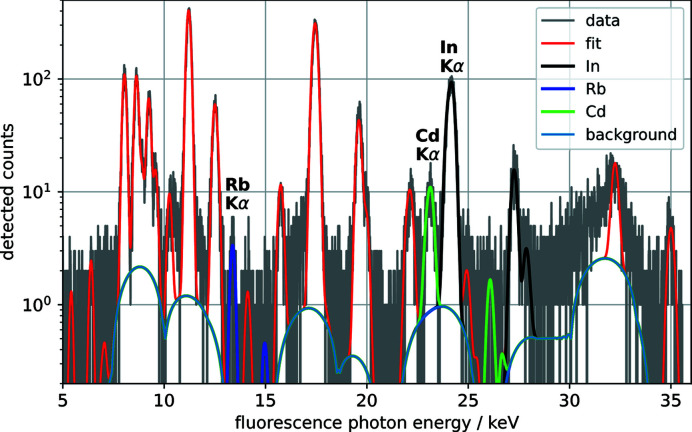
A detected and fitted fluorescence spectrum of a single scan point of the ACIGS sample. The *K*α lines of elements of particular high interest (*i.e.* Rb, Cd and In) are highlighted.

The XRF data were analyzed with *PyMca* (Heginbotham & Solé, 2017 ▸); a fit to the fluorescence spectrum of a single scan point is shown in Fig. 6 ▸ and the spatial distributions of In, Cd and Rb are shown in Fig. 5 ▸ along with the XRD peaks from ACIGS. To our knowledge, these are the first XRF maps of an ACIGS solar cell whose key elements were all assessed above their *K* absorption edge, which leads to significantly less self-absorption artefacts and more accurate quantification compared with assessment of the *L*-line XRF that strongly overlaps (Nietzold *et al.*, 2018 ▸; Ziska *et al.*, 2020 ▸). The map shows an inhomogeneous distribution of In that arises mainly from topological variations: crevices and voids, predominantly at grain boundaries, are abundant in these solar cells (Avancini *et al.*, 2018 ▸). While the CdS layer – the only layer with Cd – is supposed to be homogeneously distributed laterally for optimum electrical properties, these measurements clearly unveil inhomogeneities and suggest that the chemical-bath deposition of CdS leads to partial filling of crevices and voids. Finally, the map shows that Rb is anti-correlated with In as expected, which is in agreement with earlier measurements (Schöppe *et al.*, 2017 ▸; Plass *et al.*, 2020 ▸; Ossig *et al.*, 2022 ▸).

As the setup is readily compatible with scanning the rocking curve of individual ACIGS grains, their corresponding strain can be determined. This will allow us to correlate local elemental composition with microstructural properties of ACIGS crystallites that fulfilled the Bragg condition during a scan. The number of the latter can be increased by additional scans with the sample rotated by Φ [see Fig. 1 ▸(*b*)].

## 4. Conclusions and outlook

In conclusion, we have demonstrated µ-HRXRD at a photon energy of 35 keV, which allows one to acquire the XRD signal with micrometre spatial resolution from highly absorbing samples. We showed the feasibility of this approach with two different types of samples: martensitic steel as a powder-like example and a thin-film solar cell as a single-crystal example.

For the martensite samples we were able to image boundaries of prior austenite grains as well as the lamella-like structure of the martensite phase. The observation of induced compressive strain with different shot-peening intensities validated our method. By analyzing the deformation of Laue rings in 2D and by taking advantage of the additional degrees of rotational freedom, we will aim at the determination of the full surface stress tensor by the proposed setup, where the partial detection of Laue rings will be a challenge. This will enable us to study the impact of microscopic residual stress on short crack propagation in spring steels. In turn, this will provide a computational approach to the fracture-mechanical proof of fatigue strength in spring steels, which will be much faster and more efficient for structural-integrity assessments compared with time-consuming fatigue testing (Wildeis *et al.*, 2021 ▸).

For thin-film ACIGS solar cells, we demonstrated the simultaneous assessment of XRD-enabled characterization of the microstructure with the elemental distribution of high-*Z* materials via XRF. The readily available extension to rocking-curve imaging via 3D scans opens the possibility to correlate elemental distribution with local strain, shedding light on the mechanisms of performance reduction. In addition, the *K* absorption edges of I, *E*(I) = 33.2 keV, and Cs, *E*(Cs) = 36.0 keV, of perovskite solar cells are also accessible by increasing the photon energy to 42 keV.

Future improvements of the setup will include increasing scanning speed, as well as the utilization of smaller focus sizes. Here, total scan times were limited not by the XRD signal (several 10000 counts per scan point and diffraction peak) but by the overhead of utilized stepping positioners, which was kept to ∼100%. Thus, a reduction of scan times by at least a factor of ten is readily feasible for ‘fly scan’ capable positioners. Furthermore, it has already been demonstrated that focal spot sizes of down to 110 nm are achievable by utilizing phase plates for aberration corrections at 15 keV (Ossig *et al.*, 2021 ▸), as well as at 35 keV (Falkenberg *et al.*, 2020 ▸), at P06. Spatial resolution on the nanoscale should be enabled by the nominal angular resolution below 0.1 µrad and the nominal translation resolution of 1 nm. For future experiments, in­accuracy of angular calibration can be overcome by increasing the number of scan points for the calibration sample; hence, absolute strains will be reported. Thus, the setup will provide users from the materials-science community the means to determine micro- and nano-structures as well as the elemental composition even in highly absorbing samples.
